# The protective effect of taking care of grandchildren on elders’ mental health? Associations between changing patterns of intergenerational exchanges and the reduction of elders’ loneliness and depression between 1993 and 2007 in Taiwan

**DOI:** 10.1186/1471-2458-13-567

**Published:** 2013-06-10

**Authors:** Feng-Jen Tsai, Sandrine Motamed, André Rougemont

**Affiliations:** 1 Master program in Global Health and Development, Taipei Medical University, Taipei, Taiwan; 2 Institute of Social and Preventive Medicine, University of Geneva, Geneva, Switzerland

**Keywords:** Aging, Elder, Intergenerational exchange, Depression, Loneliness, CES-D, Mental health, Taking care of grandchildren, Study of Health and Living Status of the Middle-Aged and Elderly in Taiwan, Taiwan

## Abstract

**Background:**

The 20th century’s rapid industrialization and urbanization brought important social changes to Taiwan, including an increased number of elders living alone, which has increased risk of depression for the elderly. This study aimed to evaluate the changing pattern regarding the effect of intergenerational exchanges on elders’ depressive symptoms from 1993 to 2007.

**Methods:**

Data from the second-, fourth- and sixth-wave surveys of the *Study of Health and Living Status of the Middle-Aged and Elderly in Taiwan* were analyzed. This study collected elders’ individual sociodemographic characteristics, their self-reported health status and their intergenerational exchanges, including living with partners or with their children and their provision of care for their grandchildren. Information about elders’ depression was evaluated using the 5-item *Epidemiological Studies Depression Scale (CES-D)*.

Changes in elders’ intergenerational exchanges and depressive symptoms were compared during these study periods (chi-square test). Then, logistic regression was performed to determine how significantly elders’ intergenerational exchanges were associated with their depressive symptoms across the three years 1993, 1999 and 2007.

**Results:**

The prevalence of elders living with partners decreased from 1993 to 2007 by 19%, and that of living with their children decreased from 1993 to 2007 by 7%. Conversely, the percentage of elders providing care for grandchildren dramatically increased, from 9% in 1993 to 21% in 2007. Elderly people had significantly fewer depressive symptoms in 2007 than in 1993.

After adjusting for confounders, those living without a partner, living without children or providing no grandchild care had a greater risk of feeling lonely and being depressed. However, during the period 1993 to 2007, the impact on elders’ depression and loneliness of co-residing with a partner or with children decreased at the same time that the impact of their provision of grandchild care increased. In 2007, elders who provided no grandchild care were significantly more likely to feel lonely and sad as well as to have high CES-D scores; these strong associations were not found in 1993 and 1999.

**Conclusions:**

This study illustrates how taking care of grandchildren protects against depression and loneliness in elderly Taiwanese. We argue the need, in an aging society, for improving intergenerational interaction and recommend careful evaluation of the interaction between population policies and those of social welfare, such as child care.

## Background

The rapid aging of populations, triggered by low fertility rates and longer life expectancy, is a global phenomenon that requires a research and policy response concerning the potential consequences of this important demographic evolution [1,2]. One important issue concerning aging is depression in elderly individuals, especially loneliness caused by the evolution of intergenerational contact in a changing society [3,4].

In the 20th century, studies regarding associations between older adults’ intergenerational exchanges and their mental health mainly focused on elderly persons’ parent–child relationships and noted the importance of contingent exchanges between elders and their adult children in promoting older adults’ psychological well-being [5,6]. However, in aging societies, attention is being transferred to the impact of intergenerational exchanges between grandparents and grandchildren on elders’ mental health. As grandparents’ provision of grandchild care is central to the model of intergenerational solidarity, this provision was identified as a particularly important form of multigenerational family support [7,8].

Typical and common arrangements for grandparents’ provision of grandchild care are baby-sitting over the weekend or during the evening, caring for children when their parents are at work, or taking care of grandchildren under other circumstances on a regular or irregular basis. Previous findings, indicating that grandparents provided child care more sporadically when mothers were relatively young and worked nonstandard hours but provided extended full-time grandparent care for mothers with more extensive full-time employment, further reflect parents’ need for assistance from their own parents to take care of grandchildren in this changing society [9].

The majority of studies on the grandparent-grandchild relationship and its effect on elders’ health have emphasized that grandparents are responsible for the care of grandchildren and reported the possible risks of such caregiving on elders’ physical health and depressive symptoms [10-12]. However, a recent study has shown benefits to grandparents who take care of grandchildren, which is supported by the social exchange theory, especially from the perspective of emotional reward [13]. With the emerging nuclearization of both young and old generations brought about by modernization, the importance of grandparent-grandchild intergenerational exchanges in promoting elders’ mental health may be expected to increase, especially in eastern countries.

Taiwan is a typical Chinese society, strongly influenced by the traditional value of filial piety. It is the responsibility of family members to extend family generations and to have frequent intergenerational transfers. Chinese culture not only requires adult children to take care of their elders but also addresses the elders’ wishes and willingness to have and to take care of grandchildren [14,15]. With a decreasing fertility rate (from 1.81 children per woman in 1990 to 1.68 in 2000 and then to 1.1 in 2006) and an increasing life expectancy (from 71.6 years for men and 77.6 years for women in 1993 to 75.09 years for men and 81.90 years for women in 2007), the aged population will comprise over 20% of the overall Taiwanese population in the near future (2026) [16]. Rapid industrialization and urbanization during the 20th century have brought about a social evolution, including recognition of the government’s responsibility to offer welfare systems [16] as well as more elders living without partners and apart from their children. These changes in intergenerational exchanges and living arrangements present a high risk for loneliness in older people. Our previous study found that, as a form of support for their adult children and an alternative source of company, the prevalence of grandchild care provided by grandparents has increased over time in Taiwan [17]. This finding not only reflected the growing importance of the grandparent-grandchild relationship but also raised the issue of the mental health effects of this changing pattern on elders. Because the impact of the changing intergenerational exchange pattern on elders’ depression and loneliness has not yet been evaluated, we conducted the current study to fill that research gap.

## Methods

### Participants and survey design

This study uses data from the second-, fourth- and sixth-wave surveys of the *Study of Health and Living Status of the Middle-Aged and Elderly in Taiwan*, a longitudinal, multidisciplinary national survey representing the population of individuals aged 50 and over in Taiwan. The first-wave survey of the study was conducted in 1989 on people aged 60 and over. The subsequent surveys were conducted every three years. To maintain the representation of people aged 60 and above and to extend the elderly population to individuals aged over 50, a second cohort was included in the second survey in 1996, and a third cohort was included in the fifth survey in 2003.

Written informed consent was obtained from the participants before their participation. The research protocol was approved by the Institutional Review Board of the Bureau of Health Promotion, Department of Health, R.O.C. (Taiwan).

In this study, we used information from the second, fourth and sixth waves of the surveys, which were conducted in 1993, 1999 and 2007. A total of 3155 individuals aged 64 and over comprised the sample of the second wave survey in 1993, the sample of the fourth wave survey in 1999 included 4440 persons aged over 53, and the sample in 2007 included 4534 persons aged 54 and over. For comparability, only individuals 60 years old and older were included in our analysis. To evaluate the impact of intergenerational family transfer, including co-residence with a partner, co-residence with children and care for grandchildren, on elders’ loneliness and depression, only those elders who had grandchildren and were capable of completing the Center for Epidemiological Studies Depression Scale (CES-D scale) themselves were included in this study. In addition, if an individual participated in more than one wave of the survey, only the information from the last wave was used in the study. Overall, there were 914 elders in 1993, 1792 elders in 1999 and 2292 elders in 2007 who were included in the final analysis.

Elders’ personal characteristics, including age, gender, education, ethnic group, living place and employment status, were used in the study. In addition, information about their self-reported health status, collected with the question “how do you feel about your current health status?”, was used in the analysis. Responses were provided using a 5-point Likert scale, ranging from “very good” to “very bad”.

To evaluate elders’ intergenerational family transfers, elders were asked whether they were currently co-residing with an adult child, whether they were currently co-residing with a partner and whether they currently cared for their grandchildren to help their adult children. In the 1999 and 2007 questionnaires, the frequency of taking care of grandchildren, rated as “usual” or “sometimes”, was also collected. To compare this with the data from 1993, responses of “usual” and “sometimes” were classified as “yes” in this analysis.

The CES-D scale was used to determine elders’ depression in the study. In 1993, there were only 5 items from this scale that were evaluated in the questionnaire. These items were “I was in a bad mood”, “I felt lonely”, “People were unfriendly”, “I felt sad” and “I had no energy for things”. Responses were provided using a 4-point Likert scale, ranging from “none” to “always”. To compare the data with those from 1993, those 5 items in 1999 and 2007 were used for analysis. The internal consistency reliability of scale scores of all waves of the *Study of Health and Living Status of the Middle-Aged and Elderly in Taiwan* was confirmed in a previous study [18].

### Statistical analysis

Two approaches were used to analyze the data. For summary statistics, the chi-square test was used to compare elders’ personal characteristics, including age, gender, education, ethnic group, living place, employment status and self-reported health status, between the 1993, 1999 and 2007 samples. Elders’ intergenerational family transfers, including co-residence with partners, co-residence with their children and care for their grandchildren, as well as their loneliness and depression, as evaluated by the CES-D scale, were also compared among the 1993, 1999 and 2007 samples by chi-square test.

Each CES-D scale item was classified into two levels, either “depressed” or not; thus, responses other than “none” were classified as “depressed”. All CES-D item scores were also totaled to represent the levels of elders’ depression. The respondents in the upper tertile of CES-D scores were defined as a high-risk depressive group. Then, multiple logistic regressions were performed to determine the associations between elders’ intergenerational family transfers and their depressive symptoms for each year and for the total samples after controlling for potential confounding factors, including age, gender, education, ethnic group, living place, employment status and self-reported health status. Observations with missing data in any variable were excluded from the multivariable model.

The odds ratios (ORs), the 95% confidence intervals (CIs) and the 0.05 significance level were calculated using the software SPSS version 18.0.

## Results

### Individual characteristics and intergenerational transfer

Elders’ personal characteristics and their intergenerational family exchanges are shown in Table 1. All factors, including age, gender, education, ethnicity, living place, employment status and self-reported health status, differed significantly between three different survey years. In addition, elders’ intergenerational family exchanges, including their co-residence with partners, co-residence with their children and care for grandchildren, were all statistically significant between those three years. A total of 48% of surveyed elders were illiterate in 1993, but only 26% were illiterate in 2007. However, the percentage of city-dwelling elders increased from 47% in 1993 to 51% in 2007. Furthermore, the percentage of elders with no job increased from 85% in 1993 to 88% in 1999 but then decreased to 82% in 2007. A total of 69% of elders reported their self-perceived health as good to normal in 1993, only 61% of the sample reported their self-perceived health as good to normal in 1999, and the percentage increased to 70% in 2007.

**Table 1:** Personal characteristics of elders, including self reported health status and intergenerational exchanges

			**Year**							
	**Total**		**1993**		**1999**		**2007**			
	**n = 5070**		**n = 914**		**n = 1792**		**n = 2292**			
**Characteristics**	**n**	**%**	**n**	**%**	**n**	**%**	**n**	**%**	**chi-sq**	**p-value**
Gender										
Male	2630	51.87	510	55.80	960	53.57	1160	50.61	8.07	0.018
Female	2368	46.71	404	44.20	832	46.43	1132	49.39		
Age (years)										
60 ~ 69	1717	33.87	270	29.54	358	19.98	1089	47.51	396.53	<0.001
70 ~ 79	2243	44.24	451	49.34	1054	58.82	738	32.20		
80 ~ 89	948	18.70	182	19.91	346	19.31	420	18.32		
90 ~ 99	90	1.78	11	1.20	34	1.90	45	1.96		
Education										
Illiterate	1678	33.10	440	48.14	653	36.44	585	25.52	197.02	<0.0001
under high school	2737	53.98	359	39.28	935	52.18	1443	62.96		
College and above	261	5.15	36	3.94	81	4.52	144	6.28		
can read	318	6.27	76	8.32	122	6.81	120	5.24		
Ethnicty										
Fuchien	3374	66.55	617	67.51	1169	65.23	1588	69.28	21.97	0.001
Hakka	851	16.79	155	16.96	296	16.52	400	17.45		
Mainlander	682	13.45	118	12.91	290	16.18	274	11.95		
others	79	1.56	22	2.41	28	1.56	29	1.27		
Living place										
Large city	716	14.12	104	11.38	249	13.90	363	15.84	26.07	0.001
Big city	623	12.29	131	14.33	220	12.28	272	11.87		
Small city	1125	22.16	190	20.79	393	21.93	542	23.65		
Township	991	19.55	186	20.35	338	18.86	467	20.38		
Rural area	1543	30.43	303	33.15	592	33.04	648	28.27		
Employment status										
No job	4230	83.43	776	84.90	1582	88.28	1872	81.68	73.34	<0.0001
Full time job	593	11.70	79	8.64	167	9.32	347	15.14		
Part time job	175	3.45	59	6.46	43	2.40	73	3.18		
Self-reported health status										
good to normal	3338	65.84	630	68.93	1088	60.71	1620	70.68	47.49	<0.0001
bad to very bad	1659	32.72	283	30.96	704	39.29	672	29.32		
Intergenerational emotional exchange										
Partnership status										
living with partner	1772	34.95	432	47.26	696	38.84	644	28.10	118.67	<0.0001
living without partner	3225	63.61	482	52.74	1096	61.16	1647	71.86		
Co-residence with children										
Yes	3191	62.94	624	68.27	1164	64.96	1403	61.21	15.59	<0.0001
No	1807	35.64	290	31.73	628	35.04	889	38.79		
Take care of grandchildren										
Yes	833	16.43	83	9.08	270	15.07	480	20.94	71.34	<0.0001
No	4165	82.15	831	90.92	1522	84.93	1812	79.06		

#missing data is not shown in this table.

Regarding elders’ intergenerational exchanges, the prevalence living with partners decreased from 47% in 1993 to roughly 28% in 2007, and the prevalence of living with adult children also decreased from 68% in 1993 to 61% in 2007. After the reclassification of the responses “usual” and “sometimes” to “yes” in 1999 and 2007, the percentage of elders’ provision of grandchild care dramatically increased, from 9% in 1993 to 15% in 1999 and 21% in 2007. This trend was contrary to the trends of elders’ living with partners and with their children.

### Elders’ CES-D scale responses and their scores

Elders’ depressive symptoms, as evaluated by the CES-D scale in 1993, 1999 and 2007, are shown in Table 2. A total of 34% of elders were in a bad mood in 1993, and 32% of elders were in a bad mood in 2007. Similarly, 28% of elders felt lonely in 1993, but only 17% of elders felt lonely in 2007. Twelve percent of elders felt that others were unfriendly in 1993, whereas 7% of elders felt that others were unfriendly in 2007. In addition, 24% of elders felt sad in 1993, but only 16% of elders felt sad in 2007; 33% of elders had no energy for things in 1993, but 25% of elders felt similarly in 2007. Therefore, elders felt less lonely and less sad, had more energy for things and felt less often that others were unfriendly in 2007 compared with those who were surveyed in 1993. The differences were all statistically significant.

**Table 2:** Elders’ response in CES-D# scale and scores

			**Year**							
	**Total**		**1993**		**1999**		**2007**			
	**n = 5070**		**n = 914**		**n = 1792**		**n = 2292**			
**Items**	**n**	**%**	**n**	**%**	**n**	**%**	**n**	**%**	**chi-sq**	**p-value**
In a bad mood										
none	3317	65.42	600	65.65	1159	64.68	1558	67.98	32.66	<0.0001
a little	524	10.34	68	7.44	231	12.89	225	9.82		
sometimes	755	14.89	150	16.41	256	14.29	349	15.23		
often or always	402	7.93	96	10.50	146	8.15	160	6.98		
Lonely										
none	3905	77.02	665	72.76	1336	74.55	1904	83.07	106.87	<0.0001
a little	343	6.77	44	4.81	176	9.82	123	5.37		
sometimes	452	8.92	129	14.11	154	8.59	169	7.37		
often or always	298	5.88	76	8.32	126	7.03	96	4.19		
Unfriendly										
none	4508	88.92	805	88.07	1563	87.22	2140	93.37	52.03	<0.0001
a little	249	4.91	48	5.25	121	6.75	80	3.49		
sometimes	158	3.12	40	4.38	69	3.85	49	2.14		
often or always	83	1.64	21	2.30	39	2.18	23	1.00		
Felt sad										
none	3998	78.86	694	75.93	1379	76.95	1925	83.99	57.36	<0.0001
a little	379	7.48	62	6.78	167	9.32	150	6.54		
sometimes	402	7.93	103	11.27	152	8.48	147	6.41		
often or always	219	4.32	55	6.02	94	5.25	70	3.05		
Have no energy for things										
none	3548	69.98	613	67.07	1211	67.58	1724	75.22	51.75	<0.0001
a little	471	9.29	72	7.88	199	11.10	200	8.73		
sometimes	597	11.78	142	15.54	226	12.61	229	9.99		
often or always	382	7.53	87	9.52	156	8.71	139	6.06		
Total CES-D scores										
0 ~ 5	4243	83.69	733	80.20	1483	82.76	2027	88.44	50.53	<0.0001
6 ~ 10	561	11.07	127	13.89	223	12.44	211	9.21		
11 ~ 15	194	3.83	54	5.91	86	4.80	54	2.36		

#CES-D referred to Center for Epidemiological Studies Depression Scale.

The percentage of elders with scores higher than 5 for the sum of the CES-D scores decreased from 20% in 1993 to 12% in 2007. This difference was also statistically significant.

### Associations between elders’ intergenerational exchanges and depressive symptoms

The associations between elders’ intergenerational exchanges and their depressive symptoms are shown in Table 3. In general, elders’ intergenerational exchanges, including taking care of their grandchildren and co-residing with their children or their partners, were found to be associated with their depressive symptoms after adjusting for confounders including age, gender, living place, ethnicity, educational level, employment status, self-reported health status and years.

**Table 3:** The odds rations of elders’ intergenerational exchange and depressive symptoms by multiple logistic regression

			**Year**									
	**Total##**			**1993###**			**1999###**			**2007###**		
	**Children**	**Partner**	**Grandchildren**	**Children**	**Partner**	**Grandchildren**	**Children**	**Partner**	**Grandchildren**	**Children**	**Partner**	**Grandchildren**
Depression symptoms\intergenerational exchanges#	ORs(95% CI)	ORs(95% CI)	ORs(95% CI)	ORs(95% CI)	ORs(95% CI)	ORs(95% CI)	ORs(95% CI)	ORs(95% CI)	ORs(95% CI)	ORs(95% CI)	ORs(95% CI)	ORs(95% CI)
In a bad mood												
none	−	−	−	−	−	−	−	−	−	−	−	−
a little to always	1.06(0.93-1.21)	1.27(1.10-1.47)**	1.19(0.99-1.21)	1.10(0.79-1.53)	1.60(1.14-2.24)*	1.06(0.61-1.84)	1.23(0.98-1.53)	1.26(0.99-1.60)	1.19(0.87-1.64)	0.93(0.76-1.13)	1.16(0.93-1.45)	1.17(0.92-1.50)
Lonely												
none	−	−	−	−	−	−	−	−	−	−	−	−
a little to always	1.30(1.12-1.51)**	2.68(2.29-3.14)***	1.44(1.14-1.81)**	1.60(1.13-2.26)**	2.67(1.87-3.82)***	1.52(0.79-2.92)	1.30(1.01-1.66)*	2.94(2.26-3.81)***	1.19(0.81-1.73)	129(1.02-1.63)*	216(1.68-2.78)***	1.46(1.05-2.03)*
Unfriendly												
none	−	−	−	−	−	−	−	−	−	−	−	−
a little to always	0.96(0.78-1.18)	1.59(1.29-1.97)***	1.39(1.02-1.89)*	0.84(0.52-1.36)	1.65(1.03-2.66)*	2.83(0.97-8.31)	1.15(0.85-1.57)	1.83(1.32-2.54)***	1.15(0.73-1.84)	0.94(0.66-1.33)	1.03(0.70-1.53)	1.29(0.81-2.06)
Felt sad												
none	−	−	−	−	−	−	−	−	−	−	−	−
a little to always	0.99(0.85-1.16)	1.61(1.37-1.90)***	1.38(1.11-1.73)**	1.07(0.74-1.55)	1.86(1.28-2.70)**	1.57(0.79-3.10)	1.07(0.83-1.38)	1.79(1.37-2.33)***	1.17(0.81-1.70)	0.96(0.75-1.22)	1.18(0.91-1.55)	1.40(1.02-1.92)*
Have no energy for things												
none	−	−	−	−	−	−	−	−	−	−	−	−
a little to always	0.99(0.86-1.14)	1.15(0.98-1.33)	1.16(0.95-1.40)	0.75(0.53-1.06)	1.38(0.98-1.95)	1.42(0.79-2.55)	1.15(0.92-1.45)	1.00(0.78-1.28)	1.11(0.81-1.54)	0.98(0.79-1.22)	1.07(0.84-1.36)	1.03(0.79-1.35)
Total CES-D scores												
0 ~ 5	−	−	−	−	−	−	−	−	−	−	−	−
6 ~ 10	1.09(0.90-1.32)	1.65(1.34-2.02)***	1.30(0.98-1.72)	1.38(0.89-2.13)	1.86(1.19-2.91)**	0.83(0.40-1.73)	1.17(0.86-1.60)	1.79(1.28-2.50)**	1.10(0.69-1.74)	0.99(0.72-1.34)	1.25(0.89-1.75)	1.54(1.02-2.34)*
11 ~ 15	0.95(0.69-1.31)	2.66(1.90-3.72)***	1.95(1.12-3.43)*	0.84(0.42-1.69)	3.43(1.65-7.11)**	5.71(0.71-45.58)	1.20(0.74-1.95)	2.94(1.73-4.98)***	1.24(0.53-2.86)	0.87(0.48-1.57)	1.45(0.79-2.69)	1.78(0.77-4.16)

#children referred to five without children vs. co-resident with children, partner referred to five without partner vs co-resident with partner, grandchildren referred to not take care of grandchildren vs take care of grandchildren.

##controlling for age, gender, ethnics, educational level, employment status, living place, self-reported health status and years.

###controlling for age, gender, ethnics, educational level, employment status, living place and self-reported health status.

Not co-residing with a partner was the most important factor in developing depression in elderly people. Elders who lived without partners were more likely to exhibit depressive symptoms than those who lived with partners. Moreover, elders who did not live with their children had a significantly higher risk of feeling lonely (OR = 1.30). Taking care of their grandchildren was also significantly associated with their loneliness, sadness and the feeling that others were unfriendly. Elders who did not provide grandchild care had a greater risk of feeling lonely (OR = 1.44) or sad (OR = 1.38) or of feeling that others were unfriendly (OR = 1.39); in addition, they were more likely to have high depression scores (OR = 1.95).

Although co-residing with a partner showed strong associations with the overall CES-D scores and all CES-D scale items (except energy for things, in 1993), this effect decreased with time. In 1999, elders’ co-residence with a partner still had an effect on elders’ loneliness, impressions of unfriendliness, sadness and CES-D scores. However, a statistically significant impact of elders’ co-residence with partners was only found on elders’ feelings of loneliness (OR = 2.16) in 2007. Similarly, the impact of elders’ co-residence with their children diminished with time. Although elders’ co-residence with their children was significantly associated with their loneliness in 1993, 1999 and 2007, the odds ratio decreased from 1.60 in 1993 to 1.29 in 2007. In contrast, the impact of taking care of grandchildren on elders’ depression increased with time. Elders who did not provide grandchild care had a higher risk of feeling lonely (OR = 1.46) and sad (OR = 1.40) and had a significantly higher risk of having high CES-D scores (OR = 1.54) in 2007, but these strong associations were not found in 1993 and 1999.

## Discussion

Our study results reveal the changing pattern of associations between elders’ intergenerational exchanges and their depression and loneliness from 1993 to 2007. In general, elders had fewer depressive symptoms in 2007 than they did in 1993. In addition, elders’ intergenerational exchanges, including taking care of grandchildren, co-residing with a partner and co-residing with their children, were all found to be associated with elders’ depression and loneliness. Elders who lived with a partner or children or who provided grandchild care had a lower risk of feeling lonely and a lower risk of having depressive symptoms. However, the impact of elders’ co-residence with partners and children on their depression and loneliness decreased over time, whereas the impact of elders’ provision of grandchild care on their depression and loneliness increased over time. In 2007, elders who did not provide grandchild care had a significantly higher risk of feeling lonely and sad and were significantly more likely to have high CES-D scores; these strong associations were not found in 1993 and 1999.

The changing role of women might be one of the important factors affecting the evolution of family arrangements. The female participation rate in the labor force in Taiwan increased from 36.4% in 1960 to 49.6% in 2010 because of industrialization and urbanization, which resulted in an increased need for child care, especially from grandparents [19]. In the same period, the number of illiterate elders in Taiwan gradually decreased, and a greater number of elderly individuals lived in urban areas than in rural areas. In addition, a greater number of elders lived without partners, and fewer elders lived with their children. Furthermore, elders provided more grandchild care than before, while more of their adult children entered the work force and began having children. These changes show that the daily focus of elderly people transferred from concern for their partners and adult children to care for their grandchildren. The associations between the changing pattern of elders’ intergenerational involvement and its effect on their mental health reflect not only changing relationships within families but also the increasing impact of interacting with grandchildren on preventing depression in the elderly.

Assuming the role of a grandparent has become a normal stage in the family cycle; because of increasing life expectancy and decreasing fertility, most people now have more years to devote to their grandchildren than ever before. This phenomenon might also lead to unexpected side effects, such as healthy, wealthy grandparents competing for the attention of fewer grandchildren [20]. Our finding that grandparents who take care of grandchildren had greater psychological well-being might be a reflection of their desire to interact with their grandchildren. The emotional attachments between grandparents and grandchildren have been described as unique in that the relationship is exempt from the psycho-emotional intensity and responsibility that exist in parent–child relationships. Previous studies on the grandparent-grandchild relationship mainly focused on the perspective of the grandchild and reported children’s feelings of love and acceptance from their grandparents [21,22]. Our study results also found a positive impact of interacting with grandchildren on elders’ psychological well-being, which reflects the elderly’s need for their grandchildren’s love and company.

In addition, previous studies based on the social exchange viewpoint, in which older people feel unhappy when depending on an imbalanced exchange relationship, highlighted the correlation between elders’ greater life satisfaction and their roles as “givers” [23]. Compared with the need to be supported, the inability to establish a reciprocal relationship could more seriously devastate elders’ morale. Therefore, elders who provide more grandchild care gain emotional rewards from helping their adult children and interacting with their grandchildren.

Our participants were elders aged above 60 years old who were retired or nearly retired. Although elders today report reduced loneliness and sadness and fewer depressive symptoms, owing to advanced communication techniques and social media, most elders in this age group experience serious social losses both within and outside of the family, especially after retirement. In addition, recent studies have reported the strong correlation between elders’ loneliness and poor health outcomes, including death and multiple measures of functional decline, reflecting the fact that loneliness is an emerging issue for elders [24]. Following our study findings of the importance of grandparent-grandchild interactions in preventing elders’ loneliness, more studies are needed for a better understanding of the impact of intergenerational transfers on elders’ mental health.

There were some limitations in this study. First, our finding can only be considered to be an association (rather than a cause) because the information on elders’ intergenerational exchanges and their responses about depression and loneliness were cross-sectional. Second, we did not address the possible inverse causation that people with depression are less likely to perform grandchild care. There may be other potentially explanatory variables beyond the scope of the current study, such as poor mental health or geographic distance from children and grandchildren, that simultaneously increase depression and loneliness and reduce grandchild care. Third, the ages of the grandchildren were not specifically considered because of the lack of that information in this study. Fourth, we believe that we underestimated the impact of providing care for grandchildren on elders’ mental health in Taiwan because of the high “reporting threshold”: in Chinese culture, elders who care for grandchildren for only part of a day might not be considered as taking care of grandchildren.

## Conclusions

In conclusion, our study results demonstrate the increasing influence of grandchild care provided by grandparents on the grandparents’ depression and loneliness over time in Taiwan. Our findings indicate that elders who provided more grandchild care had an improved psychological status. However, we also recognize the possible negative effects that grandchildren can have on elders’ physical and mental health. Therefore, further studies are needed to evaluate the changes in intergenerational transfers and their effects on elders’ mental health in an aging society. In addition, we suggest careful evaluation of the link between social welfare and population policies, such as child care policies, to encourage an increase in the interactions between grandchildren and noncustodial grandparent(s). We recommend that policy makers widen the scope of particular social welfare policies to link their specific purposes with other related functions.

## Abbreviations

CES-D: Center for Epidemiological Studies Depression Scale.

## Competing interests

The authors declare that there were no competing interests in this study.

## Authors’ contributions

FJ Tsai designed the study, collected the data, performed the statistical analysis, interpreted the data, and drafted the manuscript. SM participated in formulating the study and interpreting the data and helped draft the manuscript. AR participated in formulating the study and interpreting the data and helped revise the manuscript. All authors read and approved the final manuscript.

## Authors’ information

Feng-jen Tsai holds a PhD in public health and an LLM degree in law. She is an assistant professor at the Taipei Medical University (Master program in Global Health and Development) and a lawyer in Taiwan.

Sandrine Motamed, MD, MPH is a lecturer at the University of Geneva (Medical Faculty), a chief resident at the University Hospital of Geneva and an adjunct associate professor of the University of Hokkaido, Japan.

Andre Rougemont, MD, MPH is an honorary professor and the former Director of the Institute of Social and Preventive Medicine, University of Geneva.

## Pre-publication history

The pre-publication history for this paper can be accessed here:

http://www.biomedcentral.com/1471-2458/13/567/prepub
